# Analysis of coupled coordination and spatial interaction effects between digital and tourism economy in the Yangtze River Delta region

**DOI:** 10.1371/journal.pone.0307756

**Published:** 2024-08-29

**Authors:** Zeyun Yang, Senyao Sang, Yaru Zhu

**Affiliations:** Zhejiang Ocean University, Zhoushan, Zhejiang, China; Zhongnan University of Economics and Law, CHINA

## Abstract

The coupled and coordinated development of the digital economy and tourism economy has become an inevitable choice for achieving high-quality development in both sectors. This paper proposes a conceptual index system using entropy weight method and coupling coordination degree model for analysis of the coupling coordination relationship between digital economy and tourism economy. After that, the paper introduces the Moran’s Index to examines the spatial heterogeneous effects of coupling coordination degree. This framework is applied to 41 cities in the Yangtze River Delta region during 2011–2021. The results show that the temporal changes of the coupling coordination relationship between digital economy and tourism economy in 41 cities are quite satisfactory, while the regional differences are large. Furthermore, the coupling degree between digital economy and tourism economy shows the strong spatial agglomeration effect, and the spatial proximity of regions with similar integration indicates that the "Matthew Effect" gradually highlights the clustering of high and low levels. Based on the results of analysis, this paper finally puts forward several policy recommendations to provide a referential path for the integration of digital economy and tourism.

## Introduction

With deep integration of digital technologies such as artificial intelligence, cloud computing, and blockchain, the digital economy is rapidly affecting the global production factor input structure, industrial structure, and competitive landscape [1]. The digital economy, as a new economic form brought about by digital technology and a "new driving force" for future development, has gradually become a strategic priority option for various countries’ economic policies [2]. The value of China’s digital economy reached 45.5 trillion yuan (about 6.3 trillion U.S. dollars) in 2021, accounting for 39.8 percent of the country’s GDP [3]. Impact of the digital economy on development of the national economy is essentially use of data as a new factor of production, breaking through regional and industry thresholds for economic development, and cross correlation between the digital economy and different industries such as agriculture, industry, and service [4]. The correlation is manifested not only in innovation of industry production efficiency brought about by digital technology, but also in emerging new formats or industry development trends of the digital economy itself and industry integration and sustainable development [5]. The integration between the digital economy and the tertiary industry, especially in the service industry, is increasing [3].

As an important part of the service industry, tourism industry has certain particularity in the integration and development of digital economy because of its open and diversified industrial system [6]. Tourism industry is a cluster industry complex that provides tourists with necessary consumer goods and services [7]. Development elements of the tourism economy not only need to coordinate the diverse stakeholders in tourism activities, but also need to coordinate upstream and downstream industries of tourism product production [8]. Because of this, development of tourism industry plays a dual multiplier effect for social and economic factors in sustainable development of the national economy. During the COVID-19 epidemic, online tourism products have developed rapidly based on the digital industry [9]. In 2021, the transaction scale of online tourism products has exceeded 1.3 trillion yuan [10]. The development of tourism industry will put forward higher requirements for informatization and provide financial support for informatization construction, so as to promote the improvement of informatization level [11].

From the perspective of tourist behavior, tourism decision-making essentially is an information-driven activity [12], in which tourists obtain knowledge of tourism destinations through pre-travel information resource exchange, thereby forming a destination pre-image [13]. And tourism is a highly customized and differentiated behavior, where tourists pursue personalized experiences [14]. Additionally, destinations form the public images through tourists`word of mouth, then attract potential consumers [15]. In addition, digital economy has gradually changed the development mode of the tourism industry as a whole. On the one hand, development of digital technologies such as artificial intelligence and big data has improved efficiency of tourism resource allocation, thereby improving tourism service quality and management efficiency [16]. On the other hand, digital technology such as blockchain and cloud computing have promoted derivatives of new products, formats, and models in the tourism industry, such as VR/AR tourism [17, 18], NFT digital collectibles, digital museums, and metaverse tourism. In summary, while the digital economy drives transformation and development of the tourism industry and enhances the perceived value of tourists, the tourism industry provides new opportunities and development approaches for development of the digital economy. Overall, development of digital economy and tourism economy are mutually beneficial [19]. Therefore, there is a natural fit between integration and development of the digital economy and tourism economy, not only development of digital economy reduces cost of information exchange, but also a digital platform provides experience cocreation between tourists and tourism service supplies [20, 21].

There are continuous and stable symbiotic conditions and integration basis between digital economy and tourism industry [7]. Various theories and models are employed by researches to analyze the relationship between digital economy and tourism, such as two-way fixed effects and mediating effects models [22], the comprehensive evaluation of multiple indicators [23], coordinated development theory [24], and coupling model [25]. With the increasing popularity of tourism, the relationship between tourism and economic development has been extensively studied [26–28], and for the relationship with the eco-environment [29], as well as the interactive relationship among the digital economy, technological innovation, environment and tourism [30–32]. In the era of big data, research on the relationship between digital economy and tourism development has begun to focus on the role of digital economy in sustainable tourism development strategies [33–35], and the construction and management of the smart destinations [36, 37].

The definition of coupling originated in physics and was applied to social economics afterwards [38]. which is used to describe the intensity of interactions between two or multiple systems. The coupling coordination degree model (CCDM) is a tool based on the coupling degree to reflect the intensity of cooperative development and has been widely used in empirical applications [39–41]. Considering the coupling relationship between the digital economy and tourism, the coordination of the two systems was investigated in recent years. Ma B B et al. analyzed the coordinated development of the digital economy and tourism industry for 31 provinces and regions in China’s mainland from 2011 to 2020 [7]. Shu et al. measured the coupling coordination degree and the obstacle factors of coordinated development of 31 provinces and regions in China’s mainland [42].

By considering the spatial heterogeneous effect, Ma B B et al. discussed the spatial discrepancy of the coordination between the digital economy and tourism economy for 31 provinces and regions in China’s mainland [7]. Cai C Y et al. used panel data from 284 prefecture-level and higher cities in China from 2011 to 2019 and constructed a spatial Durbin model (SDM) to empirically test the spatial effect and mechanism of the digital economy on tourism development [43]. Tang R took the social network analysis to study the spatial characteristics of digital economy and tourism high quality development in Yangtze River Delta region based on the data of 2019 [44]. These studies demonstrate the importance of the coupling and coordinated development between digital economy and tourism, but very few of them have focused on the spatiotemporal development characteristics in urban agglomerations, resulting in practical advices absent in decision making level.

The Yangtze River Delta region is not only a vibrant hub for the development of the digital economy but also the most concentrated area for tourist cities. After the official issuance of the Outline of the Development Plan for Regional Integration in the Yangtze River Delta, the Yangtze River Delta region includes three provinces and one city: Jiangsu, Zhejiang, Shanghai, and Anhui, with a total of 41 cities. In terms of the digital economy, the Yangtze River Delta region is the most vibrant and dynamic in China. The 2022 Yangtze River Delta Digital Economy Development Report confirms that in the Yangtze River Delta, the digital economy has emerged as a driving force behind the rapid development of the overall economy and society. In 2021, the GDP of the Yangtze River Delta region reached 27.6 trillion yuan, accounting for 34.1% of the national total, with the digital economy accounting for 28% of the national total [45]. In the first half of 2023, the three provinces and one city received over 1.25 billion domestic tourists, achieving a domestic tourism revenue of over 1.5 trillion yuan. The recovery speed of the tourism industry is more than 10 percentage points higher than the national average. From 2019 to 2022, the proportion of domestic tourists in the Yangtze River Delta region increased from 44.2% to 62.3%, and the proportion of domestic tourism revenue in the country increased from 63.5% to 70.2% [46].

Based on the previous studies and the characteristics of city clusters and metropolitans, this paper proposes a conceptual index system of the regional digital economy and tourism. The entropy weight method (EWM) and the coupling coordination degree model (CCDM) are employed to analyze the coupling coordination relationship between the two systems in 41 cities of the Yangtze River Delta region, China. Then the Moran’s index (the global Moran’s I and local Moran’s I) is introduced to identify the spatial heterogeneous effect of coordinated development. After that, appropriate management recommendations are put forward to facilitate the synergetic development of the digital economy and tourism economy in the Yangtze River Delta region.

## Literature review

The previous research focused on the impact of information and communication technology (ICT) on the tourism industry. ICT has accelerated the dual reshaping of the supply side and demand side of the tourism industry [47, 48]. In terms of research methods, statistical or survey data analysis is mainly used to examine the impact of ICTs on the tourism industry [49]. Adeola pointed out that the use of smart phones and the Internet in African countries has a U-shaped relationship with the development of tourism [50]. In the context of China, Yang confirmed that the increase in Internet usage is conducive to improving the quality and efficiency of tourism development [51]. However, Wang found that the disorderly construction of information infrastructure will make the development of tourism fall into the "Solow Paradox" [52]. Besides, there are some researches focusing on the role of ICT in enriching tourists’ travel experience and improving the safety of tourism payment [53, 54].

At the macro level, the digital economy can achieve high-quality development of the tourism industry by expanding the scale of the tourism market [22, 55], improving the total factor productivity of the tourism industry and the rationalization and upgrading of the industrial structure [56]. Chen and Ling took the development of the digital economy as the driven factor of the transformation of resource allocation in the tourism industry to resource sharing [57]. The instigating effect of digital economy on tourism virtual industry cluster promotes the development of tourism to present new characteristics of virtual and real interaction, thus breaking through the traditional value chain boundary [58]. However, the digital economy has also brought negative effects and intensified competition in the tourism market [59]. big data discriminatory pricing (BDDP) and digital divide brought severe challenges to tourism governance [60].

From the perspective of enterprise management, it is believed that digitalization plays a crucial role in optimizing the organizational structure of tourism enterprises and establishing information sharing across departments, thereby eliminating non- value-added activities in the business process and improving operational efficiency [10, 61]. “Big data quality” ranked as the most influential determinant of the firms’ performance in the tourism and hospitality sector [62]. Besides, the digital economy has overcome the temporal and spatial constraints of knowledge acquisition for tourism practitioners, strengthened the collective learning mechanism of the tourism industry, and thus improved the human capital quality and output performance of the tourism industry [56, 63].

There are existing literatures in China that focuses on promoting high-quality regional economic development through collaboration [56]. The spatial effects of the digital economy on tourism development have been analyzed from the perspectives of cities, provinces, and multiple regions [42, 43, 64, 65]. It is worth noting that there have been a few studies specifically focusing on the Yangtze River Delta region, and analyzing the impact of the digital economy on cultural and tourism development. For example, Tang R analyzed the spatial characteristics for the digital economy and tourism development in the Yangtze River Delta [44] Furthermore, it is found that the improvement of the development level of the digital economy promoted the high-quality development of the cultural and tourism industry, and the promotion effect had a spatial diffusion effect [66].

In short, existing research mainly focuses on the impact of ICTs on the tourism industry [67], and offers valuable insights and methodologies on the topic. However, certain areas present opportunities for further exploration. Most studies in this field tend to analyze the broader context of China, there is a lack of detailed and in-depth research specifically focusing on the representative Yangtze River Delta region [68]. Previous studies have incorporated spatial effects into the analysis framework, but there is a lack of literature considering the spatial agglomeration and synergistic effects [7, 44, 67].

Building on the above-mentioned research gaps, this paper aims to expand on three aspects. Firstly, this study specifically focuses on the representative region of the Yangtze River Delta and an constructs indicator systems to measure the digital and tourism economic development level. Additionally, this study examines the heterogeneous effects of coupling coordination degree between the digital economy and tourism economy using panel data at the city level. Moreover, spatial autocorrelation and spatial panel regression model is used to examine the spatial agglomeration and synergistic effects.

## Materials and methods

### Research design

**Fig 1:**
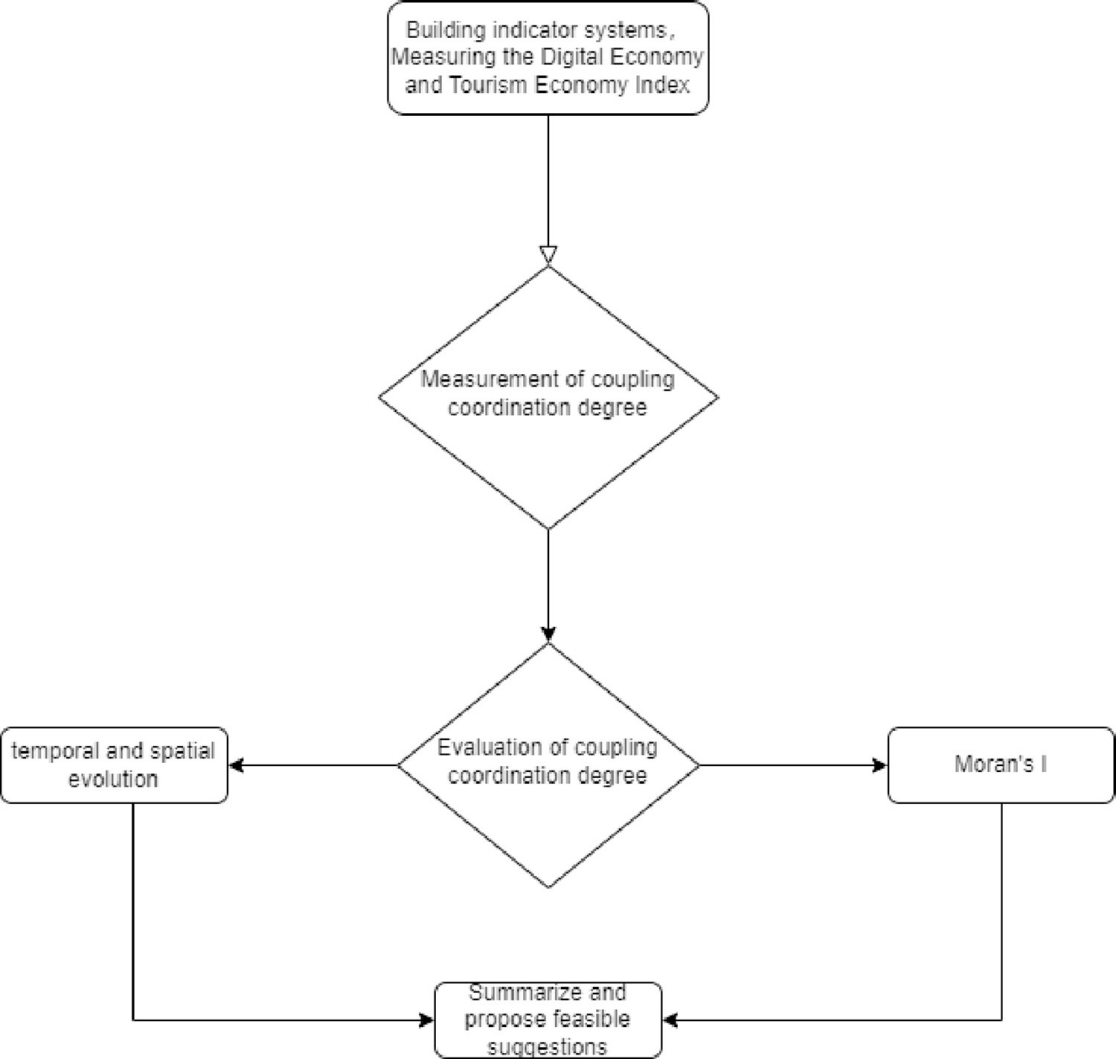
Research flowchart.

As seen from Fig 1, after the construction of the evaluation index system, the comprehensive level of the digital economy and tourism economy was obtained by using the entropy weight method (EWM). Then, the coupling coordination degree model (CCDM) was used to measure the coupling relationship between the two systems of digital economy and tourism economy in the Yangtze River Delta region, and analysis the temporal and spatial evolution. Additionally, The Moran index is employed to explain the geographical agglomeration characteristics and spatial driving effect of coordinated development of cities in the metropolitan area.

### Selection of the digital economy indicators

Following the principles of data availability, index representativeness, system correlation, and referring to relevant research results [23, 69–72]. An indicator system for measuring digital economy is constructed by five dimensions, including digital infrastructure, digital economy industry situation, digital economy development environment, digital inclusive finance, and digital penetration depth (see Table 1).

**Table 1:** The indicators of digital economy and weights.

First level indicators	Second level indicators	Directional attribute	Weight	Literature basis
Infrastructure	The number of broadband internet access users per 100 people	+	0.0516	Zhao et al. [69]
	The number of mobile phone users per 100 people	+	0.0346	Zhao et al. [69]
Digital Economy Industry Situation	Proportion of computer services and software professionals	+	0.1348	Zhao et al. [69]
	Per capita telecommunications business income	+	0.0348	Zhao et al. [69]
	Per capita postal revenue	+	0.2169	Zhao T et al. [69]
Digital Economy Development Environment	Science and technology expenditure	+	0.2898	Wang et al. [70]
Digital inclusive finance	Coverage breadth of digital inclusive finance	+	0.0299	Zhao et al. [62]
	Depth of use of digital inclusive finance	+	0.0341	Zhao et al. [62]
	Degree of digitalization of digital inclusive finance	+	0.0379	Zhao et al. [62]
Digital penetration depth	Degree of penetration of digital high-tech applications	+	0.1356	Fondeur Y & Karamé F [71], Kim N et al. [72]

Infrastructure level of the digital economy is measured by the number of broadband internet access users per 100 people and the number of mobile phone users per 100 people. Digital economy industry situation selects indicators from the internet industry, telecommunications industry, and e-commerce industry, and uses the proportion of computer services and software practitioners, per capita telecommunications business income, and per capita postal business income to characterize development of the three industries [69].

Development environment of the digital economy, mainly, refers to government policy support, represented by scientific and technological expenditures in the general public budget [70].

Digital inclusive finance draws on the China Digital Inclusive Finance Index, jointly developed by the Digital Finance Research Center of Peking University and Ant Technology Group Research Institute, and selects three dimensions. The coverage breadth of digital inclusive finance mainly includes indicators such as account penetration rate. The depth of use of digital inclusive finance mainly includes indicators related to payment services, money market fund services, credit services, insurance services, investment services, and credit services. The degree of digitalization of digital inclusive finance mainly includes indicators related to mobile access, affordability, credit access, and convenience [69].

Digital penetration depth refers to development potential of the digital economy. Taking "Baidu Index", referring to the digital economy as a proxy variable for development potential of the digital economy, annual average of daily search volume for digital economy keywords (ICT, big data, e-commerce, electronic payment, industrial internet, etc.) is collected to represent digital penetration depth variable in using python crawler technology [71, 72].

### Selection of the tourism economic indicators

An indicator system for measuring tourism economy is constructed with three dimensions: including economic contribution, industrial level, and market scale of tourism development [73–78] (see Table 2).

**Table 2:** The indicators of tourism economy and weights.

First level indicators	Second level indicators	Directional attribute	Weight	Literature basis
Economic contribution	Total tourism income	+	0.1765	Zhang H & Shi H N [73]
	Per capita tourism income	+	0.1270	Lee C C & Chang C P [74]
	The proportion of total tourism income to GDP	+	0.1098	Croes R et al. [75]
Industrial level	The proportion of employees in the tertiary industry to the total number of employees	+	0.0380	Jia J C et al. [76]
	Total number of employees in the tertiary industry	+	0.1386	Jia J C et al. [76]
	Total number of star-rated hotels	+	0.1426	Zhang H & Shi H N [73]
Market scale	Total number of tourists	+	0.1415	Zhang H & Shi H N [73]
	The proportion of total tourists to the regional population	+	0.1260	Isabel Cortés-Jiménez [77]

Economic contribution is characterized by total income of the tourism industry [73], per capita tourism income [74], and proportion of tourism total income to GDP [75]. Some missing data on total income of the tourism industry are obtained by multiplying foreign exchange income of the same year by the exchange rate between the US dollar and the Chinese yuan, and adding domestic tourism income.

Industry level, selecting relevant indicators such as number of star-rated hotels [73] and number of tourism industry employees. Due to lack of statistical caliber for data on tourism industry employees, the proportion of tertiary industry employees in the total number of employees and the total number of tertiary industry employees are used to represent the number of tourism industry employees [76].

Market scale variable is characterized by two indicators: total number of tourists [73] and proportion of tourists to population of the region at the end of the year [77].

## Methods

### Data sources

The panel data of 41 cities in the Yangtze River Delta from 2011 to 2021 were used to develop this study (see Table 3). The data used mainly comes from the following Chinese official public information: the statistical yearbooks of Zhejiang, Jiangsu and Anhui provinces and 41 cities, and statistical bulletins for national economy and social development. Data on inclusive finance measures are sourced from the Digital Finance Research Center of Peking University and Ant Group, which jointly compiled the Digital Inclusive Finance Index. And the degree data for digital penetration depth refer to the Baidu index related with digital economy. Additionally, we consult the relevant departments through telephone for data which cannot be accessed from yearbooks or communiques. Linear interpolation method is also used to calculate the missing data [79], such as the number of broadband internet access users and mobile phone users in Taizhou in 2011, Suqian in 2013, Chuzhou in 2015, etc.

**Table 3:** List of data for digital economy and tourism indicators.

Indicators	The Format of data	The sources of data
The number of broadband internet access users per 100 people	Number of broadband internet access users *100 / number of permanent residents	(1) statistical yearbook of three provinces (Anhui, Zhejiang, Jiangsu); (2) statistical yearbook of 41 cities (Anqing, Nanjing, Hangzhou, Shanghai, etc.); (3) statistical bulletin for national economy and social development of three provinces (Anhui, Zhejiang, Jiangsu); (4) statistical bulletin for national economy and social development of of 41 cities (Anqing, Nanjing, Hangzhou, Shanghai, etc.)
The number of mobile phone users per 100 people	Number of mobile phone users *100 / number of permanent residents	
Proportion of computer services and software professionals	Total number of computer services and software professionals / number of employees in the tertiary industry	
Per capita telecommunications business income	Telecommunication service income / number of permanent residents	
Per capita postal revenue	Postal revenue / number of permanent residents	
Total tourism income	Tourism foreign exchange earning and domestic tourism income	
Per capita tourism income	Total tourism income / number of tourists	
The proportion of total tourism income to GDP	Total tourism income / city’s GDP	
The proportion of employees in the tertiary industry to the total number of employees	Number of employees in the tertiary industry / number of employees	
Total number of employees in the tertiary industry	Number of employees in the tertiary industry	
Number of star-rated hotels	Number of star-rated hotels	
Total number of tourists	Number of tourists visit home and abroad	
The proportion of total tourists to the regional population	Number of tourists / city’s year-end population	
Science and technology expenditure	Science and technology expenditure	(1) statistical yearbook of three provinces (Anhui, Zhejiang, Jiangsu); (2) statistical yearbook of 41 cities (Anqing, Nanjing, Hangzhou, Shanghai, etc.)
Coverage breadth of digital inclusive finance	Coverage breadth of digital inclusive finance	Digital Inclusive Finance Index by the Digital Finance Research Center of Peking University and Ant Group
Depth of use of digital inclusive finance	Depth of use of digital inclusive finance	
Degree of digitalization of digital inclusive finance	Degree of digitalization of digital inclusive finance	
Degree of penetration of digital high-tech applications	Total daily search volume for digital economy keywords / 365	"Baidu Index" search volume for keywords related to the digital economy

### The entropy weight method (EWM)

The EWM is used to determine the weight of each indicator through information entropy, and provides a basis for the comprehensive evaluation of multiple indicators [80]. It can truly reflect the actual role of various indicators and fully utilize original data information, with avoiding the influence of human factors [81, 82]. If there are significant differences in the original data, it indicates the indicator data reflects strong differences, and it is considered the indicator has a high contribution and is given a higher weight. The calculation steps are as follows:

$$For\;positive\;indicators:\;Y_{ij}=\frac{x_{ij}-x_{ijmin}}{x_{ijmax}-x_{ijmin}}$$ (1)

$$For\;negative\;indicator:\;Y_{ij}=\frac{x_{ijmax}-x_{ij}}{x_{ijmax}-x_{ijmin}}$$ (2)

$$V_{j}=-K\sum_{j=1}^{m}Y_{ij}\ln Y_{ij},\;Y_{ij}=\frac{Z_{ij}^{\prime }}{\sum_{j}^{m}Z_{ij}^{\prime }}$$ (3)

$$U_{i=1,2,3}=\sum_{j=1}^{m}W_{ij}Z_{ij}^{\prime },W_{j}=\frac{1-V_{j}}{\sum_{j}^{m}\left(1-V_{j}\right)},\sum_{j=1}^{m}W_{ij}=1$$ (4)

Where *Y*_*ij*_ represents the proportion of indicators *j* in city *i*, *K* is a constant. *U*_*t*_ represents the comprehensive level of digital economy and tourism economy, *W*_*j*_ represents weights of *j*, and indicator weights are shown in Tables 1 and 2.

### The coupling coordination degree model (CCDM)

The relationship between systems or subsystems includes two aspects of development and coordination. Development emphasizes the evolution process of the system itself from low level to high level and from disorder to order, while coordination emphasizes the process of mutual promotion and harmonious development between system or subsystem elements. And the coupling coordination degree model can describe the development and coordination level of systems at the same time [81–83]. Then, the CCDM is used to analyze the coupling coordination relationship between digital economy and tourism economy, and the formulas are given as follows.


$$C=2\sqrt{\frac{\left(U_{1}\times U_{2}\right)}{{\left(U_{1}+U_{2}\right)}^{2}}}$$ (5)

$$T=\alpha U_{1}+\beta U_{2}$$ (6)

$$D=\sqrt{C\times T}$$ (7)

Where *C* is coupling degree of the system, *T* is coupling coordination comprehensive index, and *D* is the coupling coordination degree of two systems, *D*∈[0, 1]; *U*_*1*_ and *U*_*2*_ representing digital economy and tourism economy indices of various cities in the Yangtze River Delta region;. While *α* and *β* represent their contribution, it is assumed that *α* = *β* = 0.5 in this study considering that the digital economy and tourism are equally important [65].

According to the distribution function [83], the criteria for classifying the coupling coordination level are determined as given in Table 4.

**Table 4:** Classification of coupling coordination degree.

Serial number	Range of coupling coordination	Coordination level
1	0.00≤*D*<0.20	Serious imbalance
2	0.20≤*D*<0.30	Moderate imbalance
3	0.30≤*D*<0.40	Mild imbalance
4	0.40≤*D*<0.50	Imminent imbalance
5	0.50≤*D*<0.60	Primary coordination
6	0.60≤*D*<0.70	Intermediate coordination
7	0.70≤*D*<0.80	Good coordination
8	0.80≤*D*<1.00	Quality coordination

#### Moran’s I

The coupling and coordination between digital economy and tourism economy in the Yangtze River Delta region may not be independently distributed in space, but have significant clustering characteristics. Moran’s I is a widely used test for spatial correlation [84]. The global Moran’s I could evaluate whether the pattern of a given element and its attribute values is clustered, discrete, or random [85]. The local Moran’s I could represent the degree of correlation between its attribute values and the attribute values of surrounding units within the small range of areas, which is generally used to represent the autocorrelation properties between units [86], significantly reflect the regions where high-high or low-low clustering occurs visually [87]. In using the Moran’s index to test spatial autocorrelation of coupling coordination between digital economy and tourism economy in 41 cities, the calculation formula is as follows.


$$I=\frac{\sum_{i=1}^{n}\sum_{j=1}^{n}W_{ij}(x_{i}-\bar{x})(x_{j}-\bar{x})}{s^{2}\sum_{i=1}^{n}\sum_{j=1}^{n}W_{ij}}$$ (8)

$$I^{\prime }=\frac{x_{t}-\bar{x}}{s^{2}}\sum_{j=1}^{n}W_{ij}(x_{j}-\bar{x})$$ (9)

Where *I* is global Moran’s I while *I*’ is local Moran’s I, *I*∈[−1, 1] and I’∈[−1, 1]. When the value of *I* is positive, it indicates a positive correlation, and the closer to 1, the stronger the positive correlation, when the value of *I* is negative, it indicates a negative correlation, and the closer to -1, the stronger the negative correlation. When the value of *I* approaches 0, it indicates adjacent spatial units are not correlated; n represents the number of cities studied, *i* and *j* represents different spatial units, *x* represents coupling coordination degree, *_x* represents average of coupling coordination degrees while *s*^2^ represents variance; *W*_*ij*_ is the spatial weight matrix, if the spatial units *i* and *j* are adjacent to each other, then *W*_*ij*_ = 1, otherwise *W*_*ij*_ = 0.

## Results

### Measure of digital economy and tourism economy level

#### Measure of digital economy level

**Fig 2:**
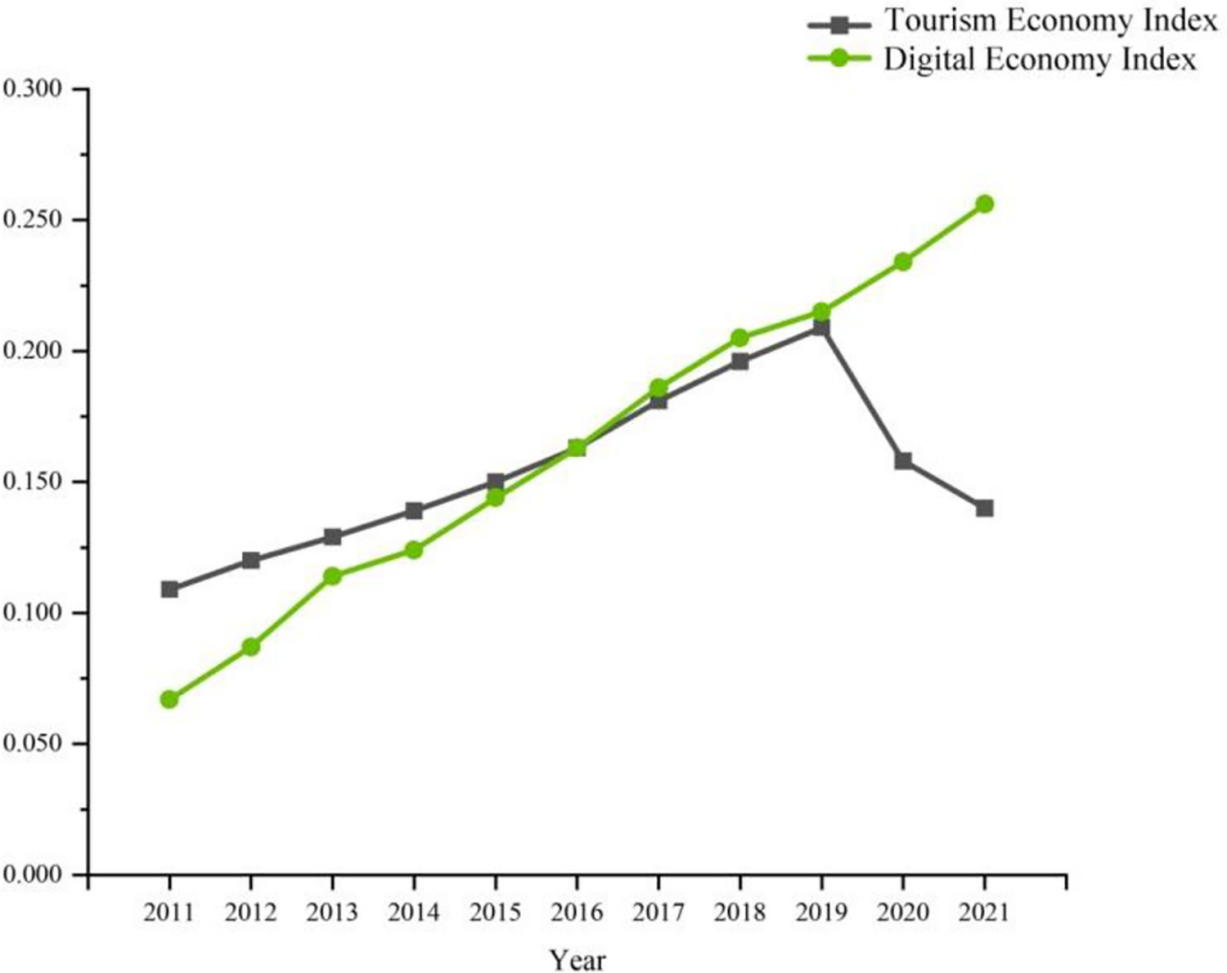
Mean of digital economy index and tourism economy index in the Yangtze River Delta region during 2011–2021.

As seen from Fig 2, the digital economy in the Yangtze River Delta region relatively lags behind the tourism economy in the early stage, but the gap between the two is gradually narrowed. In 2016, the digital economy index in the Yangtze River Delta region eventually went beyond the tourism economy index there, which indicates that, with the advancement of high and new technology in digital industry and the digital processing in the industry, the dominant period of digital development appears in the Yangtze River Delta.

The mean value of digital economy index in the Yangtze River Delta region went up to 0.256 from 0.067 in the period from 2011 till 2021, with an average annual growth rate of 14.35%. The fluctuation indicates that with the deep implementation of the development strategy of digital economy and the increase in the government investment in the field of digital economy, the advancement of digital industrialization and industrial digitization achieved significant results. However, the mean value of tourism economy index increased from 0.109 to 0.209 in 2019, reaching its peak. Then, due to COVID-19 epidemic, the tourism industry highly dependent on population mobility and social interaction was restricted, and the tourism economy index decreased to 0.140 in 2021.

Based on the analysis of the data section in 2019, 14 cities, namely, Anqing, Chizhou, Huangshan, Lu’an, Zhenjiang, Huzhou, Jiaxing, Jinhua, Lishui, Quzhou, Shaoxing, Taizhou, Wenzhou and Zhoushan, lagged behind in the digital industry, while the other 27 cities did in the tourism industry. Among the cities, the difference between tourism economy index and digital economy index of Zhoushan City was maximum, with the tourism economy index being nearly 1.4 times higher than the digital economy index. The significant gap between the two indicates that the level of local digital economy falls backward compared to highly-developed tourism industry. The cities similar to Zhoushan also include Huangshan, Huzhou and other traditional tourism cities.

**Fig 3:**
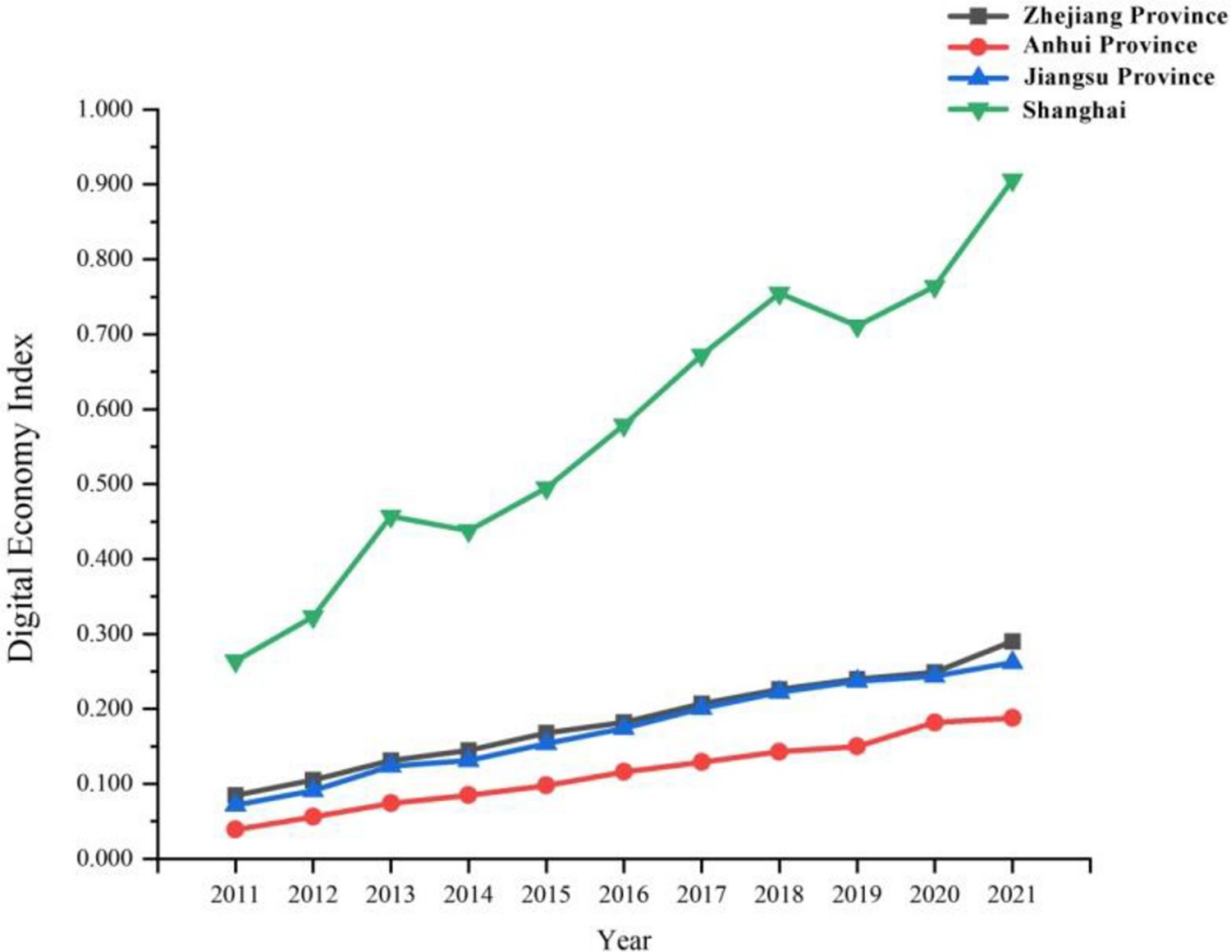
Comparison of digital economy index in Zhejiang, Anhui, Jiangsu and Shanghai during 2011–2021.

As seen from Fig 3, the mean value of digital economy index of the three provinces and one city in the Yangtze River Delta region shows a downward trend, that is, Shanghai>Zhejiang>Jiangsu>Anhui, and the broken line of the gap between the mean values in Zhejiang and Jiangsu is approximate to coincidence due to the small gap as a whole. Although the digital economy index of each province has shown a growth trend in the past 11 years, the growth rate is still slow.

#### Measure of tourism economy level

**Fig 4:**
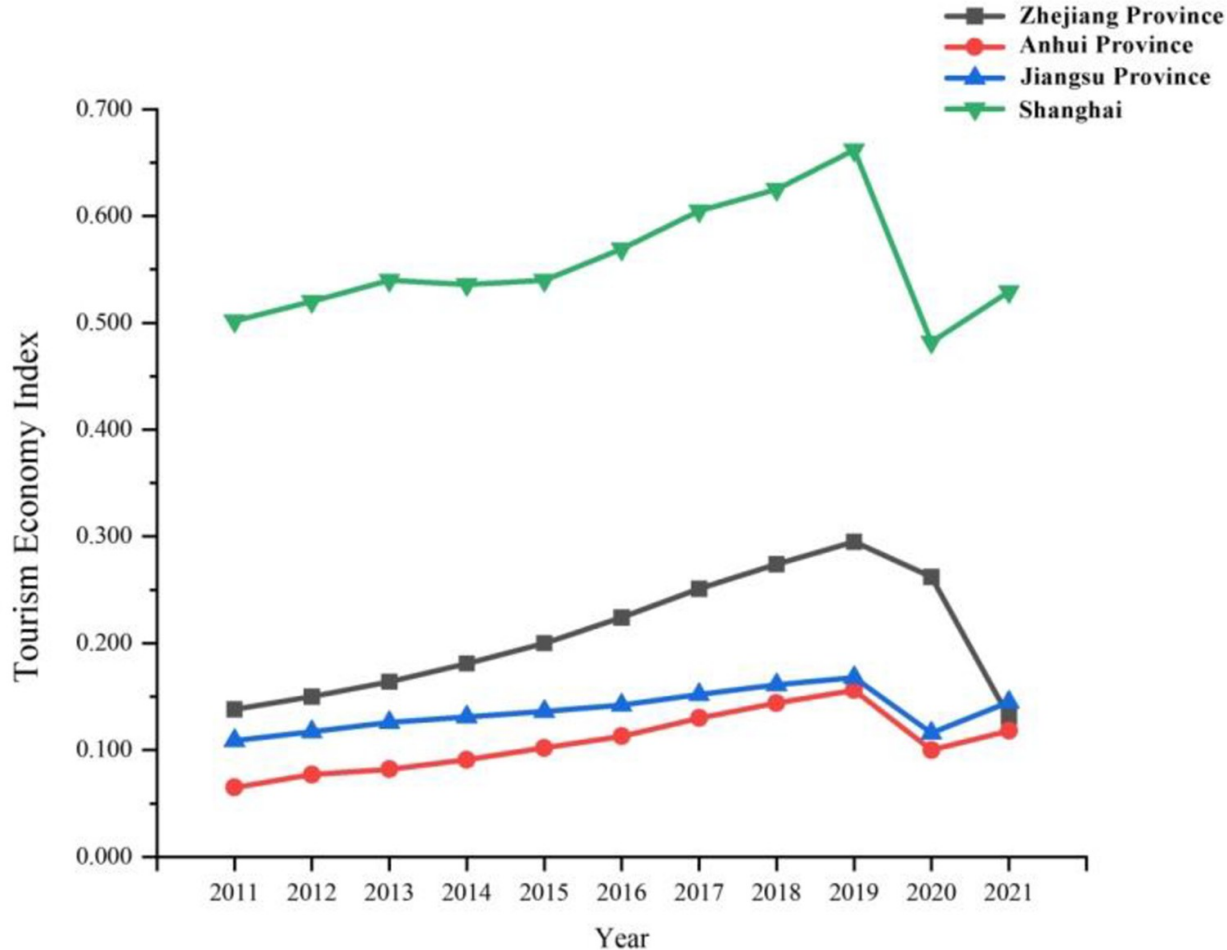
Comparison of tourism economy index in Zhejiang, Anhui, Jiangsu and Shanghai during 2011–2021.

As shown in Fig 4, the mean value of tourism economy index of the three provinces and one city in the Yangtze River Delta region shows a downward trend, that is, Shanghai>Zhejiang>Jiangsu>Anhui. The development level of tourism economy in Jiangsu Province was backward compared to its digital economy. The overall trend of tourism economy in the period from 2011 to 2021 can be divided into three stages. During the 12th Five-Year Plan period, the development of China’s tourism industry was characterized by industrialization, marketization, modernization and internationalization. The fusion of multiple formats greatly stimulated the consumption demand of the current period. The release of documents such as Opinions of the State Council on Promoting Reform and Development of the Tourism Industry effectively stimulated investment in the tourism field and pushed the tourism economy to a new level. During the 13th Five-Year Plan period, the quality requirements for tourism products were gradually improved, and so were various policies and regulations. The implementation of the development concept of "innovation, coordination, green, openness and sharing" laid the foundation for the healthy development of the tourism economy. Meanwhile, the concept of integrated development with tourism broke through various elements of the traditional tourism industry, and provided more opportunities for the sustainable development of tourism economy.

As seen from Figs 3 and 4, there is still a significant polarization (the digital economy index and tourism economy index in Shanghai are obviously higher than the other three provinces, those in Zhejiang and Jiangsu were in the middle, with a small gap between the two, while those in Anhui province are backward), although the overall development level is improved year by year and the development gap between regions is gradually narrowed. It indicates that cities with high levels of digital economy and tourism economy are concentrated in the regions with strong comprehensive strength, and there is a degree of imbalance in the digital economy and tourism economy in Jiangsu and Zhejiang. It also shows that the capacity for coordinated development within each province still needs to be improved.

### Coupling and coordinated development

#### Analysis of the temporal variation

**Fig 5:**
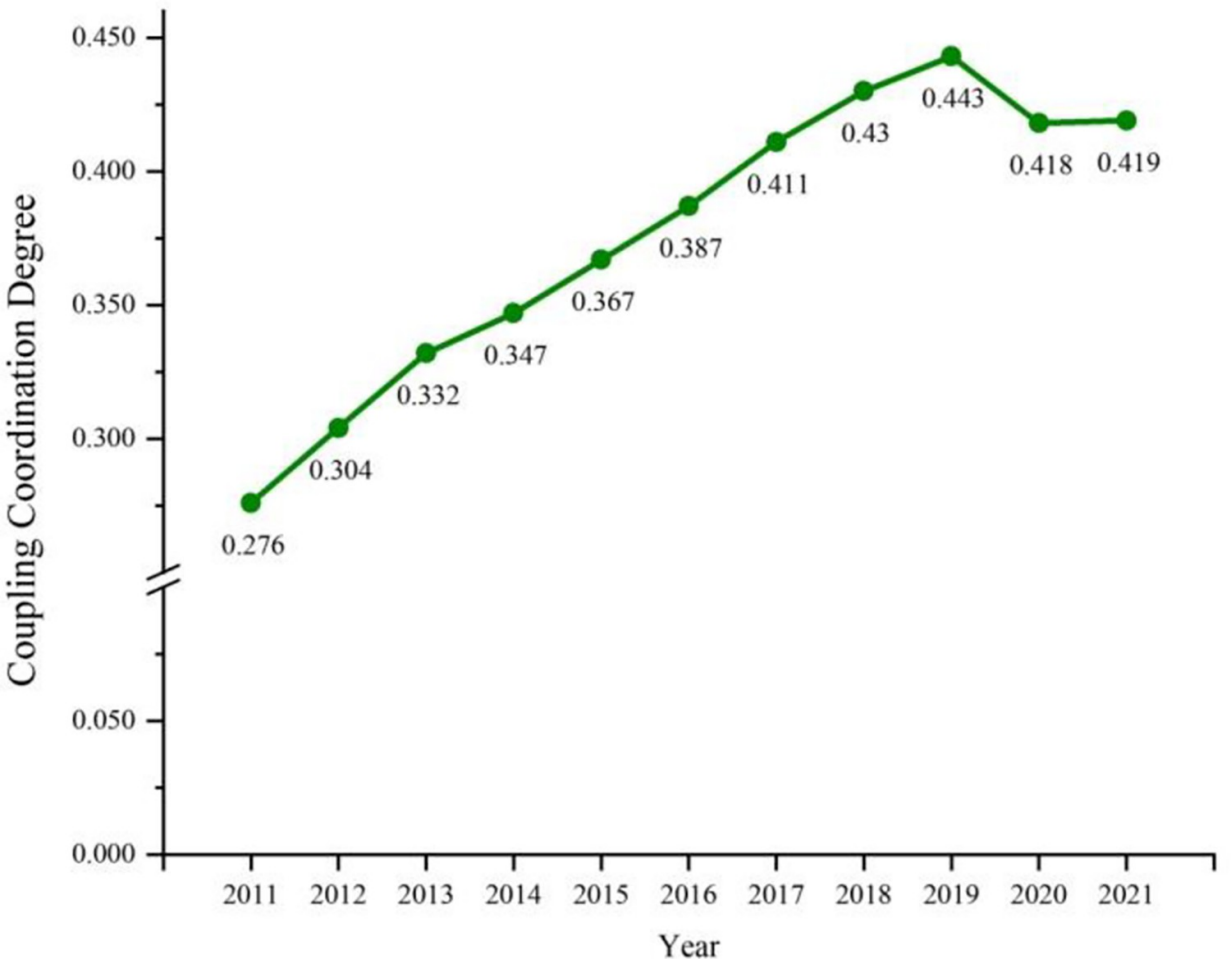
Coupling degree sequence of digital economy and tourism economy in the Yangtze River Delta region during 2011–2021.

**Fig 6:**
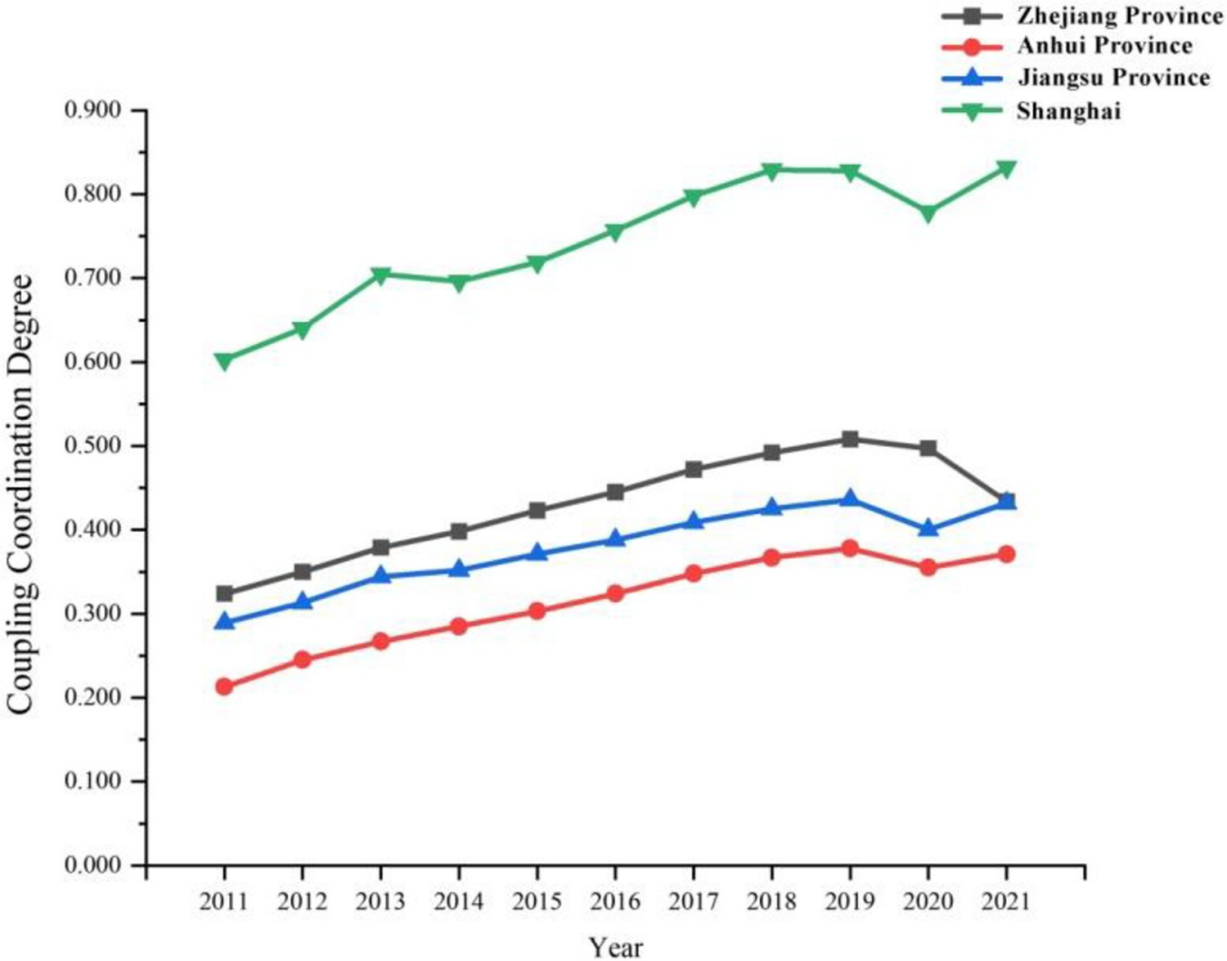
Coupling degree sequences in Zhejiang, Anhui, Jiangsu and Shanghai during 2011–2021.

As seen from Figs 5 and 6, the coupling coordination of digital economy and tourism economy in the Yangtze River Delta region varied from 0.147 to 0.832 during the period from 2011 till 2021. As seen from Fig 6, the coupling coordination of digital economy and tourism economy in Shanghai is far ahead among the three provinces and one city. The difference in the overall development level of Zhejiang and Jiangsu provinces is small, and the overall development level of Zhejiang and Jiangsu provinces is apparently better than that of Anhui. In 2011, 9 cities were in severe imbalance, 28 cities were in moderate and mild imbalance, and 3 cities were on the verge of imbalance. Only Shanghai was in moderate imbalance. During the period from 2011 to 2019, the mean value of the coupling coordination of digital economy and tourism economy in the Yangtze River Delta region steadily increased on the whole, which indicates that in the 9 years, with the growing digital innovation capability in the Yangtze River Delta region, the level of digital economy and tourism economy was significantly improved, and certain achievement was made in the coupling coordination of regional digital economy and tourism economy. Since 2020, the coupling coordination in most cities in the Yangtze River Delta region has plummeted as a result of unexpected factors such as COVID-19 epidemic and external shocks, thus leading to a decline in the mean value of regional coupling coordination: Fuyang was transitioned from mild imbalance to moderate imbalance, Nantong, Yangzhou and Xuzhou were transitioned from the verge of imbalance to mild imbalance, Lu’an was transitioned from primary coordination to mild imbalance, Wuxi was transitioned from primary coordination to the verge of imbalance. Although Nanjing and Shanghai were still in the coordination stage in 2020, the coordination level decreased by one level compared to that before the COVID-19 epidemic.

The average coupling coordination in the Yangtze River Delta region remains between 0.276 and 0.443. From the perspective of development speed, the development speed was faster during the Chinese 13th Five-Year Plan period. However, after 11 years of development, the overall coordination level of the Yangtze River Delta region was still in the range of moderate to the verge of imbalance, and did not reach the coordination level. It indicates that the coordination process between the digital economy and the tourism industry in the Yangtze River Delta region is still too slow, it is necessary to further increase the investment in digital infrastructure, stimulate new vitality of tourism talents, and make greater investment in the infrastructure of tourism industry, the development of smart tourism with intelligent scenarios, and other aspects so as to better promote the coordinated development of the two systems.

#### Analysis of the spatial pattern

**Fig 7:**
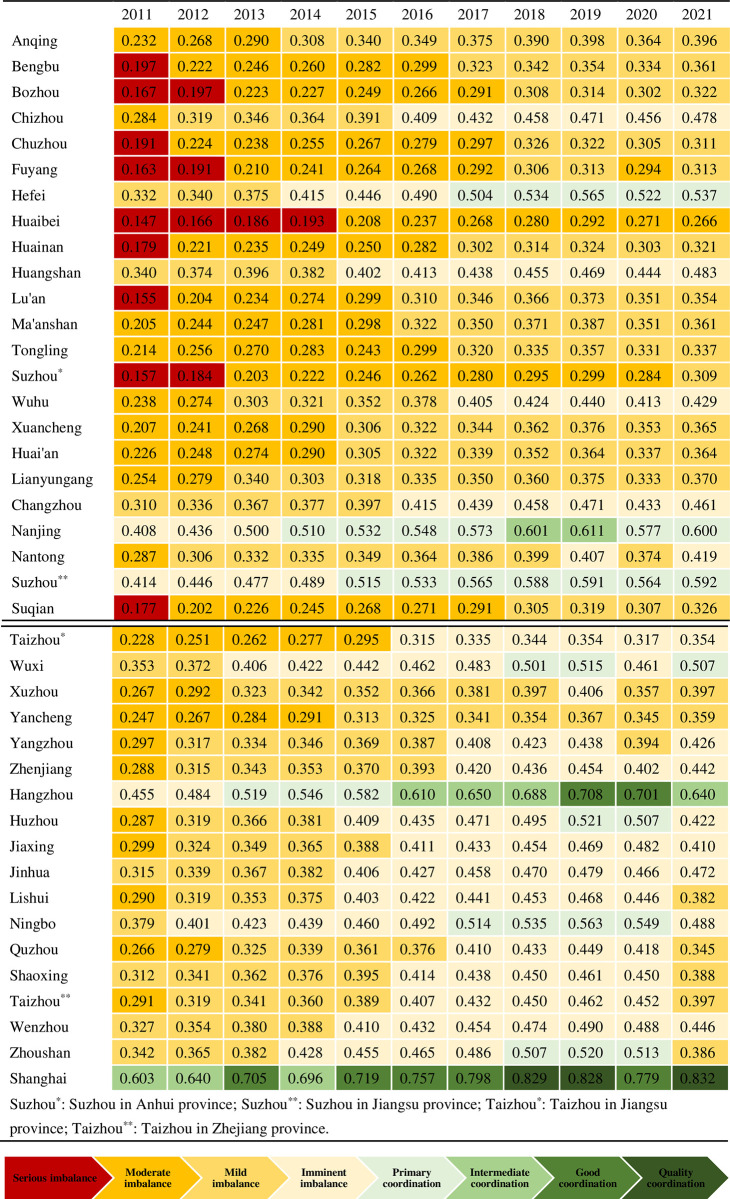
Coupling coordination level of cities in Yangtze River Delta region during 2011–2021.

Fig 7 shows the coupling coordination between digital economy and tourism economy in each city in the Yangtze River Delta region is gradually improved, the coupling coordination shows a spatial pattern of gradual attenuation from the east to the west as a whole, and from provincial capitals to other prefecture-level cities. and there is a wide gap between the east and west, with a more obvious hierarchical distribution. In each province, there is a situation that the coupling coordination of provincial capital is higher than that of other cities within the province. It indicates that the majority of provinces mainly implemented relevant strategies such as "tourism digitization" and "smart tourism" in provincial capitals compared to other cities and made remarkable achievement, and the coupling coordination in the Yangtze River Delta region was imbalance as a whole. Compared with other cities, Shanghai had a higher coupling coordination in all these years, while the level of coupling coordination between digital economy and tourism economy in most cities in Anhui Province was relatively low.

From the perspective of the entire region, the overall coupling coordination of digital economy and tourism economy in 41 cities in the Yangtze River Delta region was poor in 2011. The coupling coordination of all cities except for Shanghai were in varying degrees of imbalance, accounting for 97.5%. The case reflects that Shanghai had a higher level of digital industry and tourism industry compared to other cities. However, after four years of development, the coupling coordination of digital economy and tourism economy in Nanjing, Suzhou and Hangzhou all reached a coordinated state, with the proportion of cities in imbalance decreasing to 90.2%. Except for Ma’anshan, Tongling, Changzhou, Taizhou and Shaoxing which were still in a mild or moderate imbalance, the coordination level of all other cities increased by 1 to 2 levels after four years of development, which indicates that these cities won initial success in accelerating the development of tourism and promoting regional digital construction.

After another four years of development, the coupling coordination between digital economy and tourism economy in Hefei, Wuxi, Huzhou, Ningbo and Zhoushan changed to coordination from imbalance in 2019 on the basis of the situation in 2015, and the proportion of cities in imbalance dropped to 78%. The coordination level of other cities except Anqing, Huaibei, Huangshan, Suzhou, Xuancheng, Huai’an, Lianyungang, Suzhou, Yancheng, Jinhua, Lishui and Wenzhou increased by 1–2 levels compared to that in 2014. The coupling coordination between the tourism industry and the digital economy in the Yangtze River Delta region was further strengthened as a whole, and the proportion of the regions in moderate imbalance decreased. In 2019, the coupling coordination value of Shanghai passed 0.8, and its coupling coordination level was converted to an excellent type. Only in Nanjing, Suzhou and Wuxi of the cities in Jiangsu Province were the coupling coordination between digital economy and tourism economy in a coordinated state, and in all prefecture-level cities in Jiangsu Province were the coupling coordination levels above mild imbalance. The coupling coordination between digital economy and tourism economy reached a coordinated level in such four cities as Hangzhou, Ningbo, Zhoushan and Huzhou in Zhejiang Province, that in all other prefecture-level cities was on or above the verge of imbalance, that in Zhejiang Province was distinctly better than that in Jiangsu Province. Except for Hefei, the coupling coordination between digital economy and tourism economy in other prefecture-level cities in Anhui Province are at varying levels of imbalance, especially the coupling coordination in Huaibei and Suzhou in the northwest of Anhui Province was still in a moderate imbalance, which is in urgent need of rapid development. These regions belong to those within the province whose coupling coordination is in the low level. The poor coupling coordination indicates that the development of tourism and digital industries in the regions is insufficient, the integrated development of tourism and digital industries in these regions needs to be continuously promoted.

The impact and recovery of COVID-19 epidemic on the coupling coordination between the digital economy and tourism economy is investigated by comparing that in 2021 with that in 2019. The coupling coordination level in Suzhou increased in 2021 compared to that in 2019, in other words, it is converted to mild imbalance from moderate imbalance. The reason why the factors like COVID-19 has little effect on the fluctuation of coupling coordination may be that the development of Suzhou’s tourism and digital industry is slow. Compared to that in 2019, the coupling coordination between the digital economy and tourism economy in Nanjing and Hangzhou decreased by one level, that in Huzhou and Ningbo changed to the verge of imbalance from primary coordination, while that in Zhoushan decreased by two levels, namely, from primary coordination to mild imbalance, and the coupling coordination between the digital economy and tourism economy in Xuzhou, Lishui, Quzhou, Shaoxing and Taizhou, which were already on the verge of imbalance, decreased by a level, became mild imbalance. Compared to that in 2019, the coupling coordination level in other cities didn’t change, indicating that the tourism and digital industries were gradually improving after the impact of COVID-19 in 2020 and 2021, and a significant achievement was made in work and production resumption.

#### Analysis of synergistic development and spatial agglomeration

As seen from Table 5, the global Moran’s I index is greater than 0. As far as P value is concerned, the P values were all 0.001 from 2011 to 2020, and the Z test value were all greater than 1.96, which passed the significance test. The case indicates that the coupling coordination between digital economy and tourism economy among cities in the Yangtze River Delta region from 2011 to 2020 is not independently distributed in space, but has remarkable interdependence and correlation, that is, the city is adjacent to at least one city which has a good development in the coupling coordination between digital economy and tourism economy if its coordinated development in digital economy and tourism economy is good. Similarly, the city is adjacent to at least one city which has a poor development in the coupling coordination between digital economy and tourism economy if its coordinated development in digital economy and tourism economy is bad. Therefore, it can be inferred that the development trend and driving role of mutual promotion exist among cities in the Yangtze River Delta region, which has important reference value in the promotion of the integrated development of the Yangtze River Delta and in the reinforcement of tourism digitization and strategic decision-making of Internet plus tourism.

**Table 5:** Global Moran’s index of coupling coordination degree in 2011–2021.

Year	Moran’s I	Z test value	P value
2011	0.4041	4.6429	0.001
2012	0.3994	4.6326	0.001
2013	0.3530	4.1733	0.001
2014	0.3853	4.4626	0.001
2015	0.3729	4.2668	0.001
2016	0.3615	4.1870	0.001
2017	0.3672	4.2768	0.001
2018	0.3547	4.1554	0.001
2019	0.3563	4.1342	0.001
2020	0.4123	4.7385	0.001
2021	0.1165	1.5776	0.064

During the period from 2011 to 2019, the global Moran’s I fluctuated between 0.4041 and 0.3563 on the whole, which indicates that the spatial agglomeration effect of the coupling coordination between digital economy and tourism economy gradually decreased in these years. Since 2011, the level of the coupling coordination between digital economy and tourism economy in the Yangtze River Delta region has been evolving from a spatial agglomeration state to a random state, and regional differences have been gradually decreasing, which also meets the macro requirements of China’s recent pursuit of regional balanced development in its economy and society.

In 2020, the global Moran’s I in the Yangtze River Delta region increased sharply compared to the past two years. The reason for the sharp increase may be that the impact of COVID-19, changes in the policies on personnel mobility and the implementation of epidemic prevention and control policies via digital technology have a certain correlation with neighboring cities, thus leading to a clear agglomeration phenomenon. In 2021, its global Moran’s I was 0.1165, but the Z test value varied from -1.65 to 1.65, which indicates a random distribution of the level of the coupling coordination between digital economy and tourism economy in the Yangtze River Delta region and the absence of spatial autocorrelation. The reason for the case may be that various indicators of the digital industry and tourism industry economy in each city in the Yangtze River Delta region rapidly recovered with the introduction of a series of measures on the epidemic prevention and control, resumption of work and production and stable economy by government departments at all levels, the difference in the level of coupling coordination between digital economy and tourism economy in cities rapidly narrowed, and a random distribution state occurred once again.

In addition, global Moran’s I fluctuated in some years, for instance, in 2014. The reason for that case may be that the establishment of Shanghai Pilot Free Trade Zone enhanced the radiation effect within the region and had a profound impact on the improvement of infrastructure network and cultural exchange and fusion between cities, thereby strengthening the spatial agglomeration effect.

Local Moran scatter plot and the global Moran’s I are used to analyze the different overall distribution. The global Moran’s I is adopted to explore the spatial adjacency or similarity of neighboring units in the coupling coordination between the digital economy and the tourism economy. The local Moran scatter plot can display the spatial distribution characteristics within each region in details.

It can be found that there are more extreme points in the first and third quadrants in the local Moran scatter plot in 2011 by comparing local Moran scatter plots in 2011 and 2021 that are selected, with each point distributed in a relative scattered manner and far away from the origin, which indicates the spatial agglomeration in 2011 is relatively significant. Only Hefei’s local Moran scatter plot is located in the second and fourth quadrant in 2011, while local Moran scatter plots of Jiaxing, Xuancheng and Zhoushan were located in the second and fourth quadrant in 2021.In 2011, the cities whose local Moran scatter plot was located in the first quadrant were the regions in the south of Shanghai and Jiangsu and north of Zhejiang, such as Shanghai, Nantong, Suzhou, Shaoxing, Jiaxing and Zhoushan. The cities whose local Moran scatter plot was located in the third quadrant were the regions in the northern Jiangsu and Anhui such as Fuyang, Bozhou, Huainan, Bengbu, Huaibei, Suzhou, Xuzhou and Suqian. The case indicates there were significant spatial agglomeration in the region between northern Zhejiang and southern Jiangsu and that as well as between the norths of Jiangsu and Anhui. In 2021, the Moran scatter plot was still concentrated between the first and third quadrants as a whole, but compared to 2011, the cities whose Moran scatter plot located in the first and third quadrants were lack of Shanghai, Suzhou, Jiaxing, Zhoushan and Shaoxing. And in 2021, the distance between each Moran scatter plot was obviously shortened and there was a trend of densely drawing close to the origin, which indicates a significant weakness in spatial agglomeration in 2021.According to the analysis of the data in the two years, the number of cities belonging to high-low and low-high spatial-agglomeration regions accounts for 2.5% and 9.7% of the total sample size, respectively. The case indicates that there is a significant spatial agglomeration effect in the coupling coordination between digital economy and tourism economy in the Yangtze River Delta region, that is, the regions with high fusion level of digital economy and tourism economy are adjacent in space, while the geographical space of the regions with low fusion level of digital economy and tourism economy tend to be concentrated.

**Fig 8:**
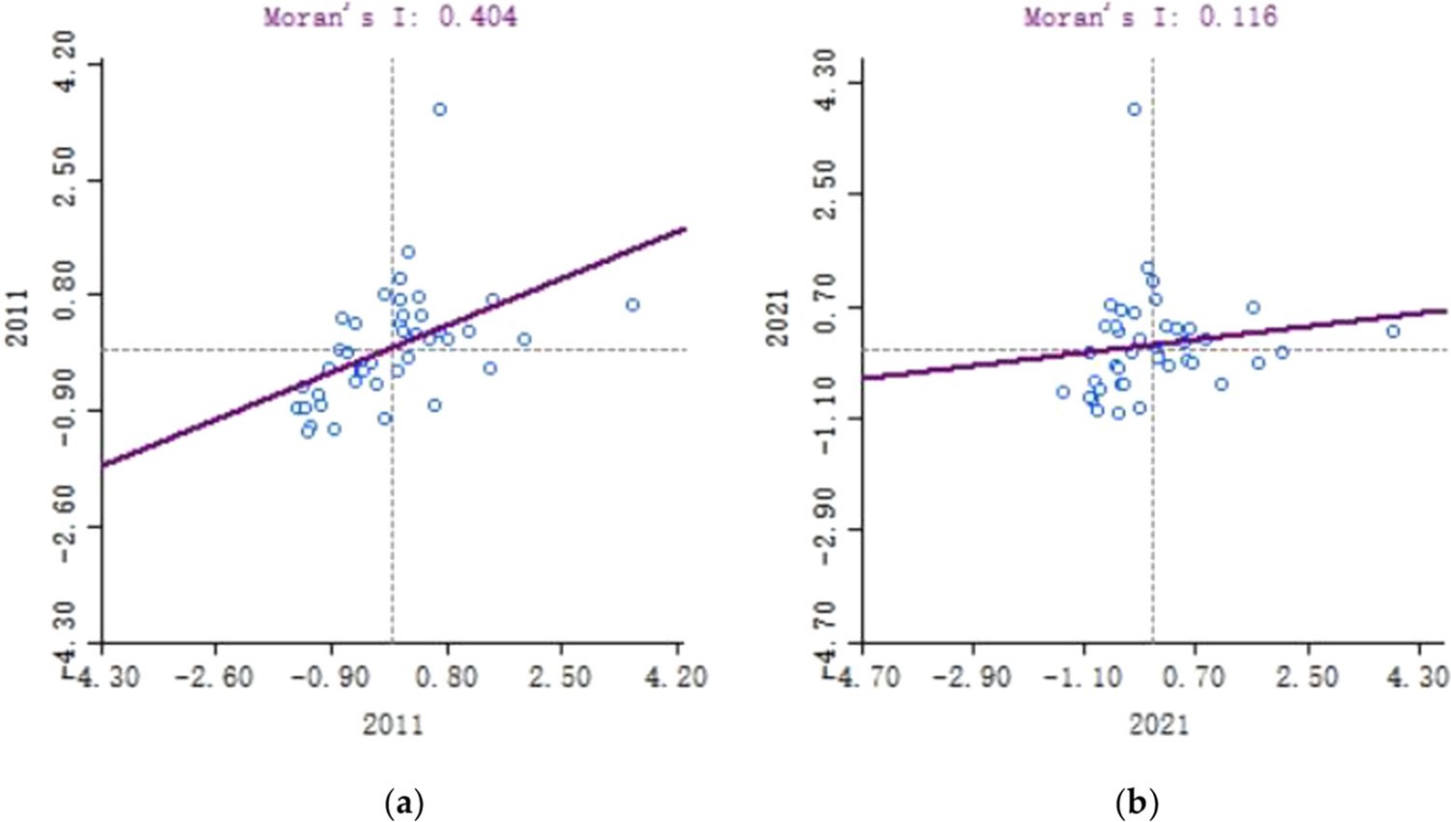
Partial Moran Scatter Map of the coupling coordination degree between digital economy and tourism economy in 2011 (a) and 2021 (b).

The spatial distribution of the fusion level of digital economy and tourism economy in the Yangtze River Delta region is further analyzed through local LISA cluster maps and significance tests. As seen from Fig 8, the fusion level of digital economy and tourism economy in the Yangtze River Delta region gradually forms two different clusters, namely, the "high-high" cluster composed of Shanghai, Nantong, Suzhou, Shaoxing, Jiaxing, and Zhoushan and the "low-low" cluster composed of Fuyang, Bozhou, Huainan, Bengbu, Huaibei, Suzhou, Xuzhou and Suqian. The marked feature of such five cites as Nantong, Suzhou, Shaoxing, Jiaxing and Zhoushan is their proximity to Shanghai, the core city in the Yangtze River Delta region. When the comprehensive digital transformation of "economy, life and governance" was promoted in Shanghai, a certain range of radiation circles also formed which not only drives the development of surrounding areas but also further stimulates the vitality in the collaborative innovation of surrounding areas. Therefore, it gradually formed a "high-high" agglomeration region with surrounding cities. The "low-low" agglomeration region composed of Fuyang, Bozhou, Huainan, Bengbu, Huaibei, Suzhou, Xuzhou and Suqian are relatively far from the core area of the Yangtze River Delta, so it belongs to the region with the growth at low speed.

According to the data from 2011 to 2020, the spatial distribution range of the "high-high" agglomeration region with coupling coordination changed a little. In 2011, the six cities in the "high-high" agglomeration region with coupling coordination were composed of Shanghai, Nantong, Suzhou, Shaoxing, Jiaxing, and Zhoushan, respectively. Huzhou was also added into the "high-high" agglomeration region with coupling coordination until 2014. The spatial distribution range of the "low-low" agglomeration region with coupling coordination slightly changed in some years, but such eight cities as Fuyang, Bozhou, Huainan, Bengbu, Huaibei, Suzhou, Xuzhou and Suqian were always in the "low-low" agglomeration region, which also reflects that the coupling coordination of tourism and digital industries in these eight cities is relatively poor.

In addition, it can be seen from the Figs 7 and 8 that there is significant heterogeneity in the fusion level of digital economy and tourism economy in the Yangtze River Delta region. In 2021, Jiaxing and Zhoushan became "low-high" agglomeration regions, which indicates that their fusion level of digital economy and tourism economy is rather low compared to places like Nantong. If driven by surrounding cities with high fusion levels, they may transition to "high-high" agglomeration region once again.

## Discussion

In the context of the construction of Digital China and the high-quality development of the tourism industry, the conclusion of this article has certain theoretical significance and practical value. Existing conclusions suggest that there is an interactive relationship between informatization and the development of the tourism industry, but further coordination is needed between the two [11]. Tang and Wang found that the improvement of the digital economy in the Yangtze River Delta has a promoting effect on the high-quality development of the cultural and tourism industry [66], and this study shows that the digital economy is not only to promote the development of the tourism economy in a one-way manner but also play a certain role in promoting the development of the digital economy, that is because the digital economy not only promotes the efficient allocation of tourism resources and improves the efficiency of tourist decision-making, but also advances the derivation of new digital formats through the development of the tourism economy, and the role that the development of the tourism industry promotes the construction of digital infrastructure shall also be given due attention. In addition, the temporal analysis of the coupling degree also demonstrates the conclusion that the integration of digital economy and tourism industry has shown dynamic evolution characteristics [7]. The perspective of spatiotemporal integration has also expanded Tang’s research on the spatial characteristics analysis in the Yangtze River Delta [44].

Analyzing their relationship from a spatial perspective is a well-established approach. Cai et al. proposed that there is a significant spatial positive correlation between the digital economy and tourism development [43]. This study incorporated the spatial effect into the analysis framework of the coupling and coordination between digital economy and tourism economy, which not only verifies the existence of integration foundations between the two systems [7], but also provides empirical evidence for the positive spatial spillover effect of the digital economy in China [43]. Based on this, this study further explored the spatial heterogeneous effect of the coupling and coordination between the two systems in the Yangtze River Delta, which provided the support for the conclusion that the improvement of the digital economy level has a spatial diffusion effect on the high-quality development of the cultural and tourism industry [66].

In summary, the research focused on the spatial differentiation of the coordinated development between digital economy and tourism economy in the Yangtze River Delta from the perspective of spatial proximity effect, not only analyzing spatial distribution, but also exploring spatial driving on the coupling. The findings show that with the promotion of the integration policy of the Yangtze River Delta, the coupling degree between digital economy and tourism economy shows the strong spatial agglomeration effect from 2011 to 2021, and the spatial proximity of regions with similar integration between digital economy and tourism economy indicates that the "Matthew Effect" gradually highlights the clustering of high and low levels. Cities with economic superiority have shown the soundly coordinated development between the two systems, and it has been found that the super city Shanghai has a positive radiation effect on the surrounding areas. Cities far away from the advantageous cities in Yangtze River Delta have a lower coupling development. In addition, cities with the good coupling can drive the transformation of the surrounding areas, while many urban agglomeration areas with poor coupling and coordination, such as in Anhui province and the junction of Anhui and Jiangsu, have remain the stagnant state from 2011 to 2021. However, this research found that under the strong promotion of policies, there may also be situations where there is no spatial correlation in the level of coupling coordination. For example, government regulatory measures after COVID-19 have rapidly reduced the difference in coordinated development, which presented the random distribution pattern, among all the cities in Yangtze River Delta region.

## Conclusions

Our case study of cities in the Yangtze River Delta region have clearly proved the effectiveness of the proposed the coupling coordination degree model and the spatial heterogeneity. And we could draw the policy implications based on the main evaluation results.

From the perspective of the development status of the two systems, the development level of the digital economy lagged behind that of the tourism economy before 2016, but the economy and tourism economy of each city in the Yangtze River Delta region during 2011–2021 shows an upward trend as a whole. The coupling and coordination relationship of the two systems is developed well, but the growth rate is slower. And it is found that although the development of the digital economy in economically developed regions is fast, it may not necessarily be coordinated with the development speed of the tourism economy in the region. On the contrary, the cities whose tourism is developed often exhibit a two-way growth at more rapid speed after being empowered by digitization, which also confirms that the coordinated development of digital economy and tourism economy relies on the foundation of the tourism industry, and digital applications are accelerators for the breakthrough of tourism economy.

The spatial heterogeneity of the coupling coordination degree between the two systems is distinct, with a distribution pattern of high in the east but low in the west, or high in provincial capitals but low in other prefecture-level cities. The development of digital economy and tourism economy shows a significant spatial positive correlation, the spatial agglomeration effect gradually declines, and the gap between cities is gradually narrowed, gradually tending to randomization and equalization. The coupling coordination between digital economy and tourism economy in the Yangtze River Delta region shows typical spatial agglomeration characteristics; The "high-high" agglomeration regions are mainly concentrated within the radiation circle of Shanghai, covering the northern regions of Zhejiang and southern regions of Jiangsu, while the "low-low" agglomeration regions are mainly concentrated in the northern regions of Jiangsu and Anhui. However. the spatiotemporal characteristics of the coupling coordination in the underdeveloped areas and the county-level units are not further explored.

Methodologically, the conceptual index system proposed in this paper has been proven as an effective tool to analyze the coupling coordination relationship between digital economy and tourism economy. Moreover, the analysis of spatial heterogeneity helps us gain a deeper understanding of the integration of digital economy and tourism in the Yangtze River Delta region. Nevertheless, due to the limited availability of data, the conceptual index system fails to fully reflect the development of the two systems in each city, which may have a certain impact on the research results.

In view of the above conclusions, the paper put forward the following policy recommendations to provide a referential path for the integration of digital economy and tourism. 1) Formulate regional differentiation strategies to give full play to the advantages of regional integration in the Yangtze River Delta region. The economically developed cities with abundant talent resources, strong scientific and technological innovation capabilities, such as the cities in northern Zhejiang, Shanghai and the cities in southern Jiangsu, shall carry forward their advantages to enhance their contributions, increase support for digital economy, continuously strengthen the construction of tourism brands and integrate digital technology with the development of the tourism industry. Cities in the central part of the Yangtze River Delta region shall act as a bridge to guide the migration of beneficial industries in coastal areas, seize the dividends of the digital economy, increase support for policies, improve the empowerment effect of digital innovation applications and unleash the potential of tourism resources. The northern Anhui and northern Jiangsu regions need to learn from the experience of the eastern coastal areas, increase the digital transformation of the tourism industry, improve their regional development strength, attract more high-quality enterprises and talents to settle, build the "industry-university-research" integration platform to adjust the structure of tourism industry, allocate resources reasonably and promote the formation of differentiated mechanisms for tourism development. 2) The support and cooperation among cities within the region shall be strengthened to promote the exchange and learning of talents, enhance the digital and intelligent knowledge reserves of tourism practitioners and promote the coordinated development of regions. In addition, the radiation and leading role of regions with high coordination between digital economy and tourism economy shall be given full play to utilize digital technology and fully tap into the potential of regional resources. Meanwhile, the importance of coordinated development between the digital economy and the tourism industry shall be emphasized to increase investment in digital facilities of tourism, promote the development of smart tourism products and lay the foundation for the coordinated development of the two. 3) In the short term, cities in the Yangtze River Delta region shall attach importance to utilizing the achievements of digital innovation to improve the efficiency of the tourism industry. In the long run, quality and quantity both shall be given consideration to. Meanwhile, attention shall be paid to introducing high-level technical talents, improving the application efficiency of digital technology in the tourism field, and ultimately achieving the goal of high-quality development of the tourism economy and a strong tourism power.

## Supporting information

S1 File (XLSX)
